# Net charge changes in the calculation of relative ligand-binding free energies via classical atomistic molecular dynamics simulation

**DOI:** 10.1002/jcc.23490

**Published:** 2013-11-19

**Authors:** Maria M Reif, Chris Oostenbrink

**Affiliations:** 1 Institute for Molecular Modeling and Simulation, University of Natural Resources and Life Sciences ViennaMuthgasse 18, 1190, Wien, Austria

**Keywords:** computer simulation, molecular dynamics, free-energy calculations, charging free energies, electrostatic artifacts

## Abstract

The calculation of binding free energies of charged species to a target molecule is a frequently encountered problem in molecular dynamics studies of (bio-)chemical thermodynamics. Many important endogenous receptor-binding molecules, enzyme substrates, or drug molecules have a nonzero net charge. Absolute binding free energies, as well as binding free energies relative to another molecule with a different net charge will be affected by artifacts due to the used effective electrostatic interaction function and associated parameters (e.g., size of the computational box). In the present study, charging contributions to binding free energies of small oligoatomic ions to a series of model host cavities functionalized with different chemical groups are calculated with classical atomistic molecular dynamics simulation. Electrostatic interactions are treated using a lattice-summation scheme or a cutoff-truncation scheme with Barker–Watts reaction-field correction, and the simulations are conducted in boxes of different edge lengths. It is illustrated that the charging free energies of the guest molecules in water and in the host strongly depend on the applied methodology and that neglect of correction terms for the artifacts introduced by the finite size of the simulated system and the use of an effective electrostatic interaction function considerably impairs the thermodynamic interpretation of guest-host interactions. Application of correction terms for the various artifacts yields consistent results for the charging contribution to binding free energies and is thus a prerequisite for the valid interpretation or prediction of experimental data via molecular dynamics simulation. Analysis and correction of electrostatic artifacts according to the scheme proposed in the present study should therefore be considered an integral part of careful free-energy calculation studies if changes in the net charge are involved. © 2013 The Authors Journal of Computational Chemistry Published by Wiley Periodicals, Inc.

## Introduction

The calculation of binding free energies is a standard task in the thermodynamic analysis of multicomponent molecular systems involving an association reaction between two system constituents, as, for example, an enzyme and a substrate, a receptor and a drug, or a nanocage and a guest compound. Physics-based approaches to compute binding free energies rely on statistical mechanics, which expresses the free energy as the natural logarithm of the system partition function (multiplied by the negative of the thermal energy,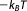, where *k*_*B*_ is Boltzmann's constant). The underlying configurational ensembles can be generated by, for example, molecular dynamics (MD) simulation. A wealth of methodological improvements, along with increased computational resources allow (in principle) the accurate calculation of binding free energies, as extensively reviewed in the case of protein-ligand association.^1^–^9^ However, if conducted without a proper eye on all potential pitfalls, binding free energies may be spuriously affected by limitations of MD simulations, such as, for example, an inadequate force-field description, approximations or/and assumptions in the free-energy calculation methodology, insufficient configurational sampling, or spurious configurational sampling due to the use of an effective electrostatic interaction function. These points are briefly discussed in turn below.

First, besides intrinsic deficiencies of classical force fields such as, for example, the neglect or mean-field treatment of electronic polarizability^10^,^11^ and the use of effective interaction energy functions^12^–^14^ with empirical parameters, additional problems arise if the system under consideration involves molecular species for which no force-field parameters are available. For instance, standard (bio-)molecular force fields may not provide parameterizations of certain metal ions, cofactors, or drug molecules. Ideally, the corresponding parameters should be parameterized against experimental data using a strategy consistent with the parameterization of the used force field. In practice, however, they are either inferred based on chemical intuition and comparison with similar compounds or taken from automatized parameterization protocols.^15^,^16^ In addition, although the solvent representation in most (bio-)molecular force fields is already highly simplistic (rigid three-site models^17^), its structural characteristics may be relinquished for the sake of computational savings, the solvent then being modeled implicitly and the solvent-generated electrostatic potential computed via numerical or empirical (generalized Born) solutions of the Poisson-Boltzmann equation.^18^–^20^

Second, because they rely on a thorough characterization of the phase space of the system, simulations involving free-energy calculations are computationally expensive, which is why a number of approximate methods are sometimes applied. For instance, the free energy of charging a neutral particle may be estimated from an electrostatic linear-response approximation^21^,^22^ or cumulant expansions at the endpoints of thermodynamic integration (TI).^23^–^25^ Similarly, the free energy of growing the van der Waals envelope of a particle is sometimes approximated using physics-based^26^,^27^ or empirical^21^ relationships. Furthermore, assumptions in the ansatz of free-energy calculation methods, such as, for example, sufficient overlap of the phase-space distribution functions in different states of relative free-energy calculations,^28^ or electrostatic linear response^22^,^29^ may limit the scope of their applicability. Lastly, discretization errors in numerical free-energy calculation methods, for example, the window width in potential of mean force calculations^30^,^31^ or the integration method in TI,^32^,^33^ limit the precision of the obtained results, although usage of optimal methods for statistical analysis [e.g., Bennett acceptance ratio (BAR)^34^,^35^ or multistate BAR^36^,^37^ approaches] may lead to significant gains in computational efficiency and statistical certainty.

Third, the phase space accessible to the system should be sampled exhaustively and according to the Gibbs measure appropriate for the desired thermodynamic ensemble, for example, canonical Boltzmann weighting in the case of simulations at constant particle number, temperature and volume. However, exhaustive sampling of phase space is complicated by the shear number of possible configurations, growing exponentially with the system size, and by energy barriers higher than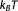, usually not amenable to transitions in plain MD simulation. Enhanced sampling methods can be used to improve coverage of the relevant phase space. A widely-used technique to address this problem involves the alteration of the potential energy function, for example, through local^38^,^39^ or nonlocal^40^,^41^ biasing, or more complex smoothening procedures,^42^,^43^ along with subsequent reweighting of the sampled configurations to the Gibbs measure corresponding to the unaltered potential energy function.

Finally, even if the phase space accessible to the system is sampled exhaustively and according to the Gibbs measure appropriate for the desired thermodynamic ensemble, the sampled configurations might not be representative of the real (experimental) situation because of an approximate or incorrect calculation of interatomic interactions. This is generally the case for electrostatic interactions which, due their long-range nature, are treated in an effective manner during MD simulations.^44^–^49^ Ensuing artifacts become strongly apparent in the configurational sampling of systems involving charged particles or in free-energy calculations involving the change of the net charge of the system (charging free energy calculations), and have been reviewed extensively.^48^–^56^ For instance, if electrostatic interactions are calculated via lattice-summation (LS) over a periodic system in charging free energy calculations, the orientational polarization of the environment of the particle to be charged will be affected by the influence of the periodic copies of this particle, which is an inappropriate contribution if actually a truly nonperiodic system is to be described. The magnitude of the introduced errors may be strongly dependent on the parameters of the system or the interaction function (e.g., the box-edge length), giving rise to so-called methodology-dependent charging free energies.^54^ It has been shown before how charging free energies of monoatomic^54^,^57^,^58^ and polyatomic^59^–^61^ ions in infinitely dilute aqueous solution can be corrected for these errors, such that methodology-independent values are obtained.

**Figure 1:**
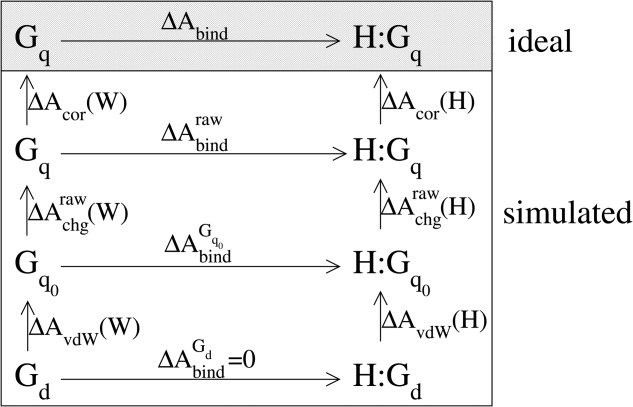
Thermodynamic cycle illustrating the connection between the binding free energy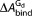 of a noninteracting (dummy) guest molecule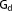, the binding free energy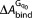 of a neutral guest molecule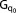, the raw binding free energy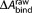 of a charged guest molecule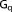 (full atomic partial charges), and the corrected binding free energy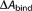 of the latter guest to a host molecule H. Corresponding complexes formed by the host and the bound guest molecules are denoted H:G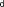, H:G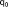, H:G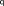, and H:G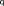, respectively. The free energies of growing the van der Waals envelope of the guest molecule,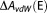, raw free energies of charging,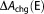, and correction terms,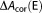, of the guest in environment E (either water W or the host molecule H) are associated with the reversible work of creating neutral van der Waals particles (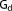 mutated into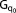), installing partial charges (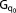 mutated into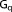) and applying corrections for approximate-electrostatics, summation, and finite-size artifacts (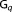 represented in the simulated situation associated with these artifacts versus in the ideal situation, i.e., a macroscopic nonperiodic system with Coulombic electrostatic interactions). The differences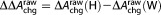 and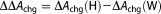 are the raw and corrected charging contributions to the binding free energy, that is, the raw and corrected binding free energies of the charged guest G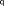 relative to its neutralized analog G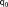 [eqs. (1) and (21)].

The goal of the present article is to address the last point above for model systems representative of a protein-ligand complex in aqueous solution, that is, to present a correction scheme for the charging of polyatomic ions in a low-dielectric cavity functionalized with different chemical groups (section “Simulated guest-host systems”), such that the raw charging free energy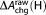 of a ligand bound to a host molecule can be corrected to a methodology-independent value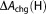 (Fig. 1). Comparison with the corresponding raw or corrected charging free energies in bulk water,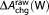 or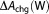, respectively, yields the raw or corrected binding free energies of the charged ligand to the host molecule relative to a neutralized analog of the ligand, in the following denoted as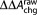 and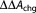, respectively (Fig. 1). The possible occurrence of methodology-dependent artifacts (caused by the use of an approximate electrostatic interaction function, an improper summation scheme and simulated systems of finite size) in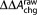 directly impairs calculations of the (absolute) binding free energy of a charged ligand and of relative binding free energies between ligands of different net charge. The value obtained for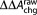 is not representative of a macroscopic nonperiodic system with Coulombic electrostatic interactions, and only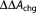 allows a meaningful comparison to or prediction of experimental data measured in systems of macroscopic extent (Fig. 1). This issue was, however, not duly appreciated in previous work. Examples from the authors' own research include, for example, the calculation of ligand binding free energies^25^,^62^,^63^ or redox potentials.^64^

On the long term, increases in computational power as currently mainly driven by graphics processing unit-based electrostatic interaction calculation^65^–^67^ and advances in multiscale simulation methodologies targeted to an improved representation of electrostatic interactions^68^,^69^ may eventually allow for the simulation of macroscopic nonperiodic systems with Coulombic electrostatic interactions, or electrostatic interactions truncated at sufficiently large distances, such that an adequate representation of experimental bulk systems is achieved. Before such techniques have become state of the art, however, a scheme that corrects for methodology-induced artifacts will prove valuable in the calculation of binding free energies of charged ligands to (bio-)macromolecular host compounds.

## Methods

### Simulated guest-host systems

**Figure 2:**
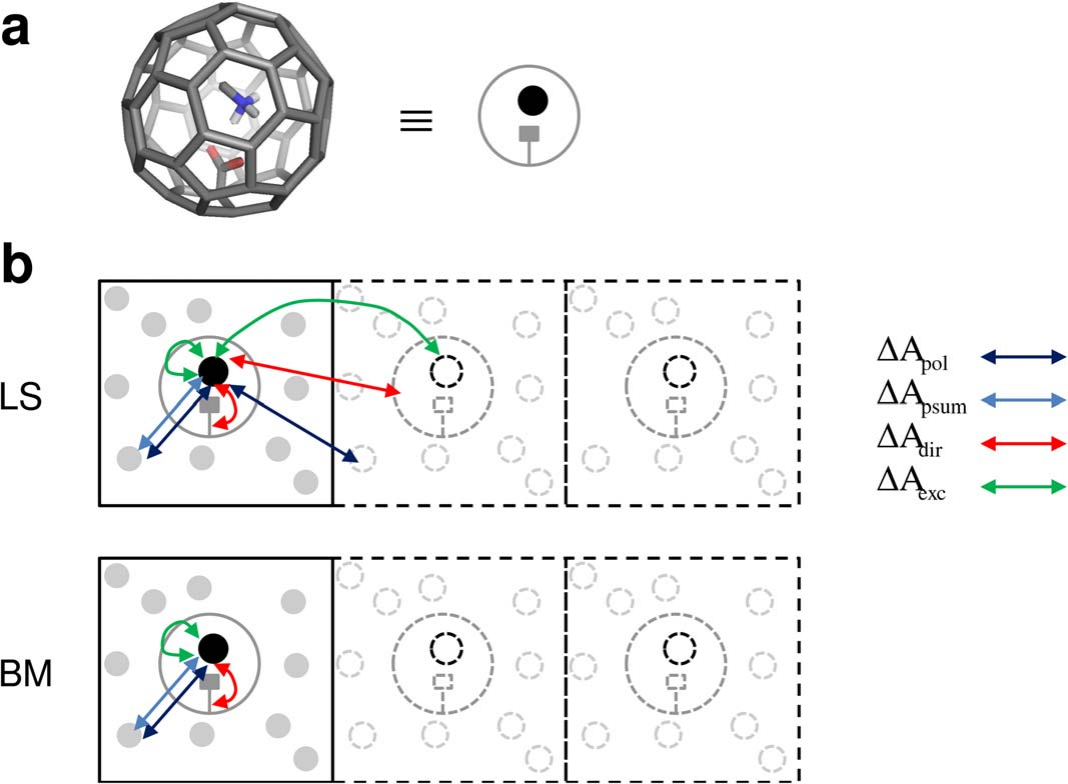
a) Stick representation of the guest-host complex MAM-CNEG. All guest-host complexes used in the present study are similarly composed of a noncovalently-bound oligoatomic ion (guest) and an artificial buckyball molecule possibly functionalized with a covalently-bound chemical group (host), as explained in section “Simulated guest-host systems.” In the following, they are depicted by a simplified schematic where the ion is drawn as a filled black circle and the buckyball is drawn as the gray surrounding structure. b) Graphic illustration of the meaning of correction terms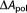,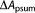,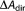, and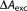 [eqs. (10), (11), (14), (15), (16), (17), (18)] to be applied to the raw charging free energy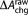 [eq. (7)], for charging of a guest molecule in a solvated host to get a corrected charging free energy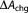 [eq. (20)] for the LS and BM electrostatic schemes. The guest, host, and water molecules are depicted in black, dark gray, and light gray colors, respectively. Periodic copies of the computational system are depicted with dashed lines. Arrows labeled with the above correction terms depict the concerned interactions, that is, guest-solvent interactions (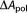,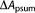), guest-host interactions (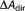), and environment-mediated guest-guest interactions (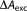). [Color figure can be viewed in the online issue, which is available at wileyonlinelibrary.com.]

A previously described^70^ simplified guest-host system was used to assess the size of methodology-induced artifacts in the calculation of (relative) charging free energies (Table1). Two oppositely-charged guest molecules, methylammonium (MAM) and acetate (ACE), binding to a host C60 molecule (buckyball), or derivatives thereof, were considered. In comparison to a realistic buckyball model, here all C-C bonds were artificially extended to 0.2 nm. For the host molecules, four variants were used: (i) an empty, apolar C60 cavity (CAPO); (ii) a C60 cavity containing a covalently-bound amide group, representing a neutral polar cavity with hydrogen-bonding capability (CHB); (iii) a C60 cavity containing a covalently-bound methylammonium group, representing a positively-charged cavity (CPOS); (iv) a C60 cavity containing a covalently-bound carboxylate group, representing a negatively-charged cavity (CNEG). Because of the high symmetry and low flexibility in these systems, the observed artifacts can be expected to be solely due to methodological aspects rather than, in addition, insufficient sampling. Figure 2a provides a graphical illustration of an example guest-host complex used in the present study. GROMOS molecular topology^71^ files for the eight guest-host complexes are provided as supporting information.

**Table 1:** Abbreviations used throughout the text for the names of guest molecules and chemical groups functionalizing the host cavity.

	Charge (*e*)	Abbreviation
Guest		
Methylammonium ion	1.0	MAM
Acetate ion	−1.0	ACE
Host functionalization		
–	0	CAPO
Formamide	0	CHB
Methylammonium	1.0	CPOS
Formate	−1.0	CNEG

The raw charging component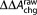 of binding free energies of charged guests MAM and ACE to host molecules CAPO, CHB, CPOS, and CNEG was calculated with MD simulation according to a thermodynamic cycle (Fig. 1) involving the free energies of guest-charging in water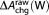 and in the host cavity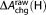,
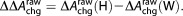
1

The MD simulations involved cubic computational boxes containing one guest molecule at multiple charge states *q*_*G*_ varying from 0.0 to the full charges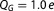 or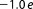 for MAM and ACE, respectively, in either pure water or the hydrated host cavity. Each system was simulated in four different box sizes of edge lengths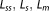, and *L*_*l*_, of about 2.46–2.53, 2.90, 3.21–3.25, or 3.80–3.81 nm, respectively, differing by the number of water molecules. Furthermore, two different methods were used to calculate electrostatic interactions (section “MD simulations”), namely either LS^72^,^73^ or molecule-based cutoff truncation in combination with a Barker–Watts reaction-field correction^74^ (BM). Simulations with the LS scheme were performed in boxes of edge length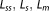, and *L*_*l*_, and are in the following referred to as LS,ss, LS,s, LS,m, and LS,l, respectively. Simulations with the BM scheme were performed in boxes of edge length *L*_*m*_ and *L*_*l*_, and are in the following referred to as BM,m and BM,l, respectively.

### MD simulations

All MD simulations were performed either with a modified version of the GROMOS96 program^71^ or with the GROMOS11 program.^75^ The former was exclusively used for free-energy calculations in simulations using the particle-particle-particle-mesh (P^3^M) method^72^,^73^ for the treatment of electrostatic interactions. Water was represented by means of the three-site simple point charge (SPC) model.^76^ Host and guest molecules were described with the GROMOS 53A6 force-field parameter set as in the previous study of Ref.^70^. For CNEG, the appropriate GROMOS improper dihedral type 1 (reference value of 0°) was used for the improper dihedral angle in the formate group rather than an erroneous type 2 (reference value of 35° as in Ref.^70^).

All simulations were carried out under periodic boundary conditions (PBC) based on cubic computational boxes. The equations of motion were integrated using the leap-frog scheme^77^ with a timestep of 2 fs. The solute bond-length distances and the rigidity of the water molecules were enforced by application of the SHAKE algorithm^78^ with a relative geometric tolerance of 10^−4^. The center of mass translation of the computational box was removed every 2 ps. The temperature was maintained at 300 K by weak coupling to a heat bath^79^ using a coupling time of 0.1 ps and the volume was kept constant. Electrostatic interactions were either calculated using LS based on the P^3^M algorithm with tinfoil boundary conditions,^72^,^73^ or using the BM scheme.^74^ The LS scheme was applied with^72^,^80^,^81^ a spherical hat charge-shaping function of width 1.0 nm, a triangular shaped cloud assignment function, a finite-difference (FD) scheme of order two and a grid spacing of about 0.08–0.12 nm in the different systems. The self-energy term^23^,^73^,^82^–^84^ of the guest molecule was not included in the electrostatic potential at the guest atom sites to be consistent with the previously developed correction scheme for single-ion solvation free energies.^54^,^57^ In simulations with the LS scheme, Lennard–Jones interactions were truncated at an atom-based cutoff distance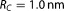. Both real-space electrostatic and Lennard–Jones interactions were calculated at each timestep based on a pairlist updated at each timestep. The BM scheme was applied with a value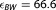 for the relative permittivity of the dielectric continuum surrounding the cutoff sphere, as appropriate^85^ for the SPC water model. Here, too, the self-energy term^84^,^86^ of the guest molecule was not included in the calculation of the electrostatic potential at the guest atom sites to be consistent with previous work.^54^,^57^ In simulations with the BM scheme, electrostatic and Lennard–Jones (van der Waals) interactions were truncated at a charge-group cutoff distance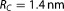, and calculated at each timestep based on a pairlist that was updated at each timestep.

The calculation of the charging component to the binding free energy was performed in two TI procedures^87^ considering the free energies of charging the guest molecules in water and in the host cavity (Fig. 1). All production simulations for the free-energy calculations were preceded by an equilibration period of 0.3 ns and lasted 1 ns. The configurations sampled along these simulations were written to file every 0.3 ps for subsequent analysis, whereas the corresponding energetic data was written every timestep.

### Structural properties

The configurations sampled in simulations LS,ss, LS,l, and BM,l of the fully charged guests MAM and ACE in hydrated host molecules CAPO, CHB, CPOS, and CNEG were analyzed in terms of the solvent radial distribution function *g*(*r*) around the buckyball center of mass, and the solvent radial polarization *P*(*r*) around the charged guest. These functions were calculated as
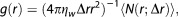
2and
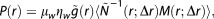
3where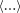 denotes ensemble averaging,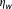 is the solvent number density,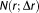 is the number of water molecules *j* for which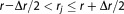 (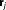 denoting all possible minimum-image vectors connecting the center of mass of the 60 buckyball carbon atoms to the oxygen atom of any periodic copy of water molecule *j*),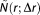 is the number of water molecules *j* for which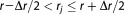 (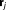 here denoting all possible minimum-image vectors connecting the MAM nitrogen atom or the ACE carboxylate carbon atom to the oxygen atom of any periodic copy of water molecule *j*),
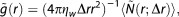
4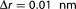 is the bin width,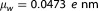 is the molecular dipole moment of the SPC water model^76^ and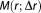 is defined as
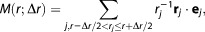
5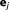 being a unit vector along the dipole moment of molecule *j*. For systems MAM-CAPO, MAM-CHB, MAM-CPOS, ACE-CAPO, ACE-CHB, and ACE-CNEG, *P*(*r*) was compared to a radial continuum-electrostatics analog, here approximated by the Born polarization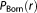 around a charge of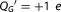 (MAM-CAPO, MAM-CHB),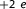 (MAM-CPOS),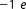 (ACE-CAPO, ACE-CHB), or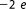 (ACE-CNEG) centered at the MAM nitrogen or the ACE carboxylate carbon atom,
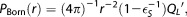
6where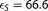 is the relative dielectric permittivity of the SPC water model.^85^ Equation (6) is an approximation because in the considered systems the charge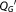 is actually spread out over multiple atom sites. In addition, the dielectric permittivity around the highly-charged MAM-CPOS and ACE-CNEG systems may be lower than the bulk value of 66.6.

### Free-energy calculations

#### Raw charging free energy

Raw charging free energies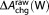 and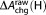 [eq. (1)] were calculated with the TI approach^87^ along progressive installation of λ-dependent intermolecular electrostatic interactions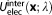,
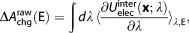
7where the subscript E denotes the environment of the guest (either W or H),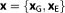 denotes the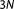-dimensional coordinate vector of the system containing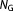 guest atoms and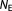 water and host atoms, λ denotes the scaling parameter of the TI procedure, and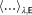 denotes ensemble averaging over configurations sampled during a simulation where the guest is in environment E, and guest partial atom charges are scaled by λ. The term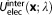 in eq. (7) was calculated based on the sampled configurations as
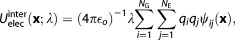
8where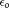 is the permittivity of vacuum and *q*_*i*_ is the partial charge of atom *i*.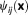 is the effective pairwise electrostatic interaction function which describes the implementation of the particular electrostatics scheme.^71^ The exact details of this function depend on the implementation in a simulation program, and can be found elsewhere.^71^,^86^ Note that the electrostatic interaction energy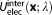 is exempt of intramolecular contributions.

During the simulations of a given charge state, the partial charges of the guest atoms were scaled by λ. For all free-energy estimates, eleven charge states (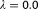, 0.1, …, 0.9, 1.0) were used and, the integral in eq. (7) was evaluated numerically with the trapezoidal rule. Statistical error estimates on ensemble averages pertaining to particular λ-values were obtained from block averaging.^88^ Errors on the free-energy values were calculated by numerical integration (trapezoidal rule) of the individual errors and amounted to about 0.2–1.9 kJ mol^−1^.

#### Free-energy correction terms

The raw charging free energies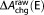 [eq. (7)] were used to calculate corresponding methodology-independent values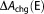 as^54^,^57^,^58^
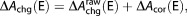
9where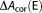 is a free-energy correction for the various methodology-dependent errors committed during the simulation. These errors have been discussed extensively for the case of monoatomic^54^,^56^,^58^ and polyatomic^61^ single-ion hydration. In simulations with the LS and BM schemes, they arise from:

- The deviation of the solvent polarization around the charged solute from the polarization in an ideal macroscopic system with Coulombic electrostatic interactions. This is a consequence of the use of a finite (microscopic) system during the simulation, for example, a computational box simulated under PBC, of the use of approximate (non-Coulombic in the limit of infinite system sizes) electrostatic interaction functions, for example, involving cutoff truncation, and of the use of a solvent model with an inaccurate dielectric permittivity. The corresponding correction term is here denoted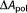. Note that in previous work,^61^ this correction term was denoted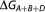, or split up into three terms^54^,^57^,^58^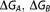, and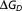.
- The deviation of the solvent-generated electrostatic potential at the atom sites of the charged solute as calculated from the simulated trajectory from the “correct” electrostatic potential. This is a consequence of the possible application of an inappropriate summation scheme for the contributions of individual solvent atomic charges to this potential, that is, summing over individual point charges (“P-summation”^89^) versus summing over charges within individual solvent molecules (“M-summation”^89^), as well as of the possible presence of a constant offset in this potential, due to the presence of an interfacial potential at the surface of the solute along with the constraint of vanishing average potential in the LS scheme. The corresponding correction term is here denoted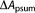. Note that in previous work,^54^,^57^,^58^,^61^ this correction term was denoted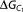.
- The spurious calculation of guest-host interactions with an effective electrostatics scheme rather than with Coulombic electrostatic interactions. The corresponding correction term is denoted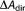. Note that it is only pertinent to systems containing the host moiety, that is, it only occurs in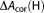.
- The presence of electrostatic interactions between excluded atoms in the guest molecule. The corresponding correction term is denoted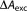. Note that it is absent in the case of monoatomic guest compounds and that it was not explicitly listed in Ref.^61^, because there these interactions were considered an integral part of the environment-induced electrostatic potential at the solute atoms.

Figure 2b illustrates the interactions addressed by the afore mentioned correction terms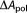,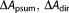, and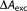 for the case of a guest molecule binding noncovalently to a solvated host. In the following, the calculation of these terms is explained.

As in previous work,^61^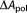 can be deduced from the results of two continuum-electrostatics calculations,
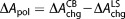
10for the LS scheme and
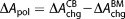
11for the BM scheme, where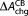 is the charging free energy of the guest molecule in a macroscopic nonperiodic system with Coulombic electrostatic interactions based on the experimental solvent permittivity (CB).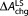 and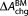 are the charging free energies of the guest molecule in a periodic system with LS or BM electrostatic interactions based on the model solvent permittivity, respectively. Here, the relative dielectric permittivity is set to 66.6 in the calculation of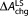 and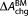 as appropriate for the SPC water model^85^ and to 78.4 in the calculation of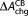, as appropriate for water,^56^ to account for the inaccurate dielectric permittivity of the SPC water model. As the guest molecules considered in this study are essentially rigid,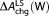 and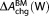 are essentially configuration-independent. Also, the rotational and translational sampling of the guest molecules in the host cavities does not lead to significant fluctuations in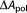 (data not shown). Therefore, the calculations of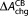,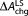, and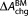 were only performed based on a single structure, taken as the final solute configuration of the simulation at guest charge states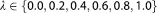 in the system with box-edge length *L*_*l*_, as
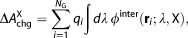
12where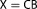, LS, or BM, and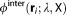 is the environment-generated electrostatic potential evaluated at guest atom site *i* and charge state λ for the given boundary conditions and electrostatics scheme X. For both the LS and BM scheme,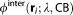 was evaluated using the FD Poisson equation solver of Refs.^90^–^92^ with the appropriate boundary conditions, and solvent permittivity. The FD solver was also used to evaluate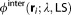. Because the FD solver does not offer the option of using the BM scheme, a combination with the fast fourier transform (FFT) Poisson equation solver of Refs.^93^–^94^ was used to evaluate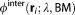 as
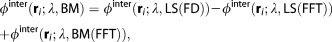
13where the first and the last two terms on the right-hand side are calculated based on the FD and FFT Poisson equation solvers, respectively. This is done to enhance cancellation of grid-discretization and boundary-smoothing errors in the two different Poisson equation solvers. In both algorithms, the grid spacing was set to about 0.02 nm and the convergence threshold for the electrostatic free energy was set to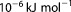. A van der Waals envelope was used to define the guest-host system, where the atomic radii were based on distances at the minimum of the Lennard–Jones potential between the different solute atoms and the oxygen atom of a SPC water molecule^76^ using the Lennard–Jones interaction parameters of the GROMOS 53A6 force-field parameter set,^95^ reduced by an estimate^96^ for the radius of a water molecule (0.14 nm). Polar hydrogen atoms were treated differently^53^,^55^ and assigned an atomic radius of 0.05 nm. Instead of using eq. (12) to evaluate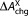, less computationally intensive but more approximate approaches can be used, which are discussed in Appendix section “*Calculation of*
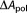
”.

The term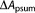 corrects for the atom-based summation scheme implied by the LS and BM schemes in comparison to a proper molecule-based summation scheme.^89^ In the present study, it is calculated as
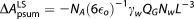
14for the LS scheme and
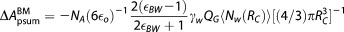
15for the BM scheme, where *N*_*A*_ is Avogadro's constant,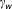 is the quadrupole-moment trace of the water model relative to its single van der Waals interaction site, *N*_*w*_ is the number of water molecules,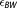 the reaction-field permittivity, *R*_*C*_ the cutoff distance, *L*^3^ the box volume (here, a constant box-edge length *L* is adopted because all simulations were performed at constant volume), and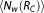 is the average number of water molecules present within the cutoff sphere around the center of mass of the 60 buckyball carbon atoms for in-host charging, or around the MAM nitrogen or ACE carboxylate carbon atoms in the case of in-water charging. For the SPC water model,^76^,^89^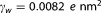. Equation (15) differs from the equation used in Ref.^61^ for the calculation of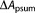. The corresponding equation used in Ref.^61^, as well a derivation of eq. (15) and a comparison in terms of root-mean-square deviations of corrected charging free energies are reported in Appendix section “*Calculation of*
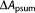
”.

The additional contribution to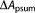 related to the quadrupole-moment trace of the guest and host molecules is implicitly accounted for through the presence of these molecules in the continuum-electrostatics calculations [eq. (12)], because the boundary conditions in the FD Poisson solver calculations under PBC enforce zero average electrostatic potential over the computational box, which is equivalent to the situation in the MD simulations. On the contrary, in the FD Poisson solver calculations under nonperiodic boundary conditions (NPBC), there is no such shifting of the average electrostatic potential. That means that the correction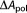, which involves a difference between calculations under NPBC and PBC [eqs. (10) and (11)] also corrects for the spurious vanishing average (over the computational box) electrostatic potential contribution due to the guest and host quadrupole moments in the MD simulations. Note that this implicit inclusion of the solute-associated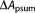 correction in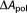 was not recognized in previous work.^61^

Similar to Ref.^61^, the term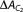 was neglected in the present study because this term is proportional to the ratio of the guest volume to the box volume, that is, its magnitude is very small for the systems considered here.

The term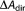, to be applied only to raw charging free energies of the guest in the host, was calculated as
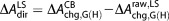
16and
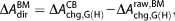
17where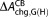,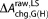, and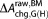 are charging free energies of the guest due to the host calculated with Coulombic electrostatic interactions in a nonperiodic system and with effective electrostatic interactions (LS or BM) under PBC, respectively. The guest-host complex configurations sampled in the explicit-water MD simulations at all guest charge states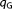 (section “*Raw charging free energy*”) were used to extract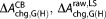, and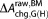 in postanalyses under NPBC (guest-host complex in vacuum described with Coulombic electrostatic interactions) and under PBC (guest-host complex described with LS or BM electrostatic interactions), respectively (section “Solute and solvent contributions to the free energy of charging”).

The term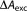 was calculated as
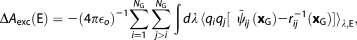
18where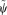 is a modified LS or BM electrostatic interaction function exempt of self term. The integrand of eq. (18) corresponds to minus the first term in eq. (20) of Ref.^61^ and corrects for the presence of electrostatic interactions between excluded atoms (first and second covalently-bound neighbors) in the Hamiltonian used in the present simulations. Electrostatic interactions between excluded atoms are equal to the normal interaction function applied to nonexcluded atoms, but reduced by the Coulombic contribution. As a result, excluded atoms may be viewed to interact via a term that depends on the representation of the surroundings of the solute, that is, periodic copies of the computational box in the case of the LS scheme and a continuous medium of homogeneous relative dielectric permittivity in the case of the BM scheme. Therefore, in the present study, interactions between excluded atoms are regarded as contributing in a methodologically-dependent way to the charging free energy. Application of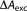 removes the corresponding contribution.

Given the above correction terms, the charging free energy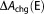 is calculated according to eq. (9) as the sum of the raw charging free energy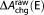, and the correction terms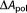 [eqs. (10) and (11)],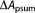 [eqs. (14) and (15)],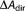 [eqs. (16) and (17)] and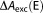 [eq. (18)] as
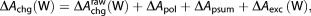
19in the case of charging in water, and as
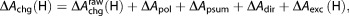
20in the case of charging in the host. These charging free energies were calculated for guests MAM and ACE in water and host molecules CAPO, CHB, CPOS, and CNEG and yield the corrected charging component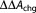 of binding free energies of the guest to the host,
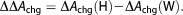
21

### Solute and solvent contributions to the free energy of charging

The trajectories of guest-host complexes were reanalyzed to obtain the raw free energy of charging the guest molecule due to the host and periodic host copies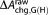 as
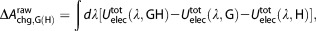
22where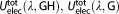, and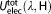 are the total electrostatic energies sampled in trajectories pertaining to guest charge states defined by λ using modified interaction parameters with full guest and host charges, full guest and zeroed host charges and zeroed guest and full host charges, respectively. The corresponding raw free energies of charging the guest molecule due to the solvent and periodic solvent copies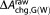 are calculated as
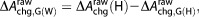
23where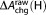 is given by eq. (7). Corrected values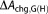 and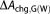 are calculated as the sum of the raw charging free energies and the correction term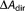 [eqs. (16) and (17)],
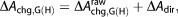
24in the case of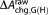, and as the sum of the raw charging free energies and the correction terms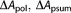, and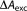 [eqs. (10), (11), (14), (15), and (18)],
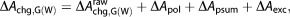
25in the case of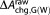.

## Results

Application of correction terms for spurious solvent polarization and wrong dielectric permittivity of the solvent model (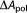) [eqs. (10) and (11)], improper electrostatic potential summation (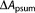) [eqs. (14) and (15)], effective guest-host direct electrostatic interactions (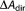) [eqs. (16) and (17)] and the presence of electrostatic interactions between excluded solute atoms in the Hamiltonian (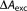) [eq. (18)] to raw charging free energies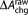 [eq. (7)] yields corrected values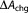 [eqs. (19) and (20)] reported in Table2 for charging of guests MAM and ACE in water or in complex with the hydrated host molecules CAPO, CHB, CPOS, or CNEG (Table1). This is illustrated for four different sizes of the computational box (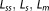 and *L*_*l*_) and the two different electrostatics schemes (LS and BM) considered (section “MD simulations”). While the root-mean-square deviations for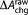 are 6.6, 6.8, 6.7, 8.1, and 9.0 kJ mol^−1^ for charging of MAM in CAPO, CHB, CPOS, CNEG, and water, respectively, and 12.4, 12.3, 11.8, 13.9, and 10.2 kJ mol^−1^ for charging of ACE in CAPO, CHB, CPOS, CNEG, and water, respectively, they are reduced to 0.8, 1.0, 1.5, 2.1, and 1.4 kJ mol^−1^ for MAM and 0.5, 0.6, 1.6, 2.5, and 0.6 kJ mol^−1^ for ACE in corresponding corrected free energies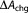. Complexes MAM-CNEG and ACE-CNEG exhibit the largest rmsd values in corrected charging free energies (2.1 and 2.5 kJ mol^−1^). Notably, for these complexes, the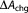 value from simulation LS,ss has higher magnitudes by up to 6.0 kJ mol^−1^ (MAM-CNEG) or up to 8.0 kJ mol^−1^ (ACE-CNEG) compared to simulations LS,s, LS,m, LS,l, BM,m, and BM,l. These deviations might be due to the inability of the continuum-electrostatics representation to capture short-range artifacts in solvent structure.

**Table 2:** Charging free energies 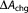 of the guest molecules MAM and ACE in hydrated host molecules CAPO, CHB, CPOS, and CNEG, as well as in water, computed in cubic computational boxes of edge length *L* containing *N*_*w*_ water molecules using LS or BM electrostatic interactions (sections “Simulated guest-host systems“ and ”MD simulations”). Values obtained with the LS scheme in boxes of edge lengths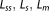, and *L*_*l*_ are labeled LS,ss, LS,s, LS,m, and LS,l, respectively, and values obtained with the BM scheme in boxes of edge lengths *L*_*m*_ and *L*_*l*_ are labeled BM,m and BM,l, respectively. The charging free energy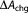 [eqs. (19) and (20)] is calculated as a sum of the raw charging free energy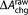 [eq. (7)], and the correction terms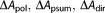, and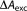 [eqs. (10), (11), (14), (15), (16), (17), (18)]. Error estimates on the raw charging free energies refer to the statistical uncertainty obtained from block averaging.^88^

Guest	Host/water	Scheme	*N*_*w*_	*L* (nm)	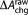 (kJ mol^−1^)	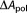 (kJ mol^−1^)	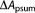 (kJ mol^−1^)	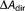 (kJ mol^−1^)	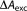 (kJ mol^−1^)	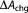 (kJ mol^−1^)
MAM	CAPO	LS,ss	471	2.46	−4.4± 0.2	−71.2	−75.4	0.0	−0.2	−151.2
		LS,s	780	2.90	−12.4± 0.2	−62.4	−76.3	0.0	−0.1	−151.2
		LS,m	1095	3.24	−17.6± 0.2	−56.8	−76.9	0.0	−0.1	−151.4
		LS,l	1792	3.81	−24.7± 0.2	−49.2	−77.1	0.0	−0.1	−151.1
		BM,m	1095	3.24	−8.5± 0.2	−72.3	−68.9	0.0	−0.3	−150.0
		BM,l	1792	3.81	−9.7± 0.2	−71.3	−67.8	0.0	−0.3	−149.1
	CHB	LS,ss	469	2.46	−38.7± 0.8	−70.5	−75.4	−0.7	−0.2	−185.5
		LS,s	780	2.90	−47.3± 0.9	−61.8	−76.3	−0.4	−0.1	−185.9
		LS,m	1095	3.24	−53.2± 0.8	−56.3	−77.1	−0.3	−0.1	−187.0
		LS,l	1781	3.81	−59.7± 0.9	−48.9	−77.3	−0.2	−0.1	−186.2
		BM,m	1095	3.24	−44.1± 0.3	−71.2	−69.0	−0.9	−0.3	−185.5
		BM,l	1781	3.80	−44.3± 0.3	−70.2	−68.0	−0.9	−0.3	−183.7
	CPOS	LS,ss	473	2.46	115.7± 0.5	−209.7	−75.4	155.8	−0.2	−13.8
		LS,s	780	2.90	111.2± 0.5	−184.6	−76.3	133.3	−0.1	−16.5
		LS,m	1090	3.23	107.6± 0.6	−168.6	−76.9	120.0	−0.1	−18.0
		LS,l	1772	3.80	102.8± 0.6	−146.4	−77.1	102.6	−0.1	−18.2
		BM,m	1090	3.23	122.2± 0.5	−212.8	−69.1	142.4	−0.3	−17.6
		BM,l	1772	3.80	119.7± 0.6	−209.9	−68.2	142.4	−0.3	−16.3
	CNEG	LS,ss	473	2.46	−234.2± 1.8	69.9	−75.4	−157.9	−0.2	−397.8
		LS,s	780	2.90	−245.4± 1.8	61.5	−76.3	−134.6	−0.1	−394.9
		LS,m	1095	3.25	−252.6± 1.6	56.0	−76.5	−120.5	−0.1	−393.7
		LS,l	1785	3.81	−261.2± 1.9	48.6	−77.2	−102.9	−0.1	−392.8
		BM,m	1095	3.25	−248.9± 0.4	70.8	−68.3	−145.1	−0.3	−391.8
		BM,l	1785	3.81	−248.7± 0.4	69.8	−67.7	−145.1	−0.3	−392.0
	Water	LS,ss	533	2.54	−172.6± 0.5	−75.8	−77.8	0.0	−0.2	−326.4
		LS,s	800	2.90	−181.8± 0.5	−66.7	−78.3	0.0	−0.1	−326.9
		LS,m	1091	3.21	−188.1± 0.5	−60.4	−78.4	0.0	−0.1	−327.0
		LS,l	1827	3.80	−197.8± 0.5	−51.3	−79.2	0.0	−0.1	−328.4
		BM,m	1091	3.21	−173.7± 0.5	−77.4	−76.6	0.0	−0.3	−328.0
		BM,l	1827	3.80	−175.5± 0.5	−75.7	−79.2	0.0	−0.3	−330.7
ACE	CAPO	LS,ss	468	2.46	−74.5± 0.2	−70.8	75.4	0.0	−0.3	−70.2
		LS,s	780	2.90	−84.5± 0.2	−62.1	76.3	0.0	−0.2	−70.5
		LS,m	1095	3.24	−90.8± 0.3	−56.5	76.8	0.0	−0.1	−70.6
		LS,l	1792	3.81	−99.1± 0.3	−49.0	77.1	0.0	−0.1	−71.1
		BM,m	1095	3.24	−65.9± 0.3	−71.7	68.4	0.0	−0.4	−69.6
		BM,l	1792	3.81	−66.2± 0.3	−70.7	67.4	0.0	−0.4	−69.9
	CHB	LS,ss	471	2.46	−117.8± 0.5	−70.2	75.4	−0.2	−0.3	−113.1
		LS,s	780	2.90	−128.0± 0.6	−61.7	76.3	−0.1	−0.2	−113.7
		LS,m	1090	3.24	−134.1± 0.6	−56.3	76.9	−0.1	−0.1	−113.7
		LS,l	1784	3.81	−142.7± 0.6	−48.8	77.2	−0.1	−0.1	−114.5
		BM,m	1090	3.24	−109.5± 0.4	−71.0	68.5	−0.3	−0.3	−112.6
		BM,l	1784	3.81	−110.0± 0.4	−70.0	67.6	−0.3	−0.3	−113.0
	CPOS	LS,ss	474	2.47	−288.7± 0.3	70.0	75.5	−157.6	−0.3	−301.1
		LS,s	780	2.90	−301.9± 0.3	61.6	76.3	−134.5	−0.2	−298.7
		LS,m	1095	3.24	−310.1± 0.3	56.1	76.5	−120.5	−0.1	−298.1
		LS,l	1781	3.80	−319.8± 0.3	48.8	77.3	−103.0	−0.1	−296.8
		BM,m	1095	3.24	−290.5± 0.3	70.8	68.3	−144.8	−0.3	−296.5
		BM,l	1781	3.80	−289.3± 0.3	69.8	67.9	−144.9	−0.3	−296.8
	CNEG	LS,ss	479	2.47	37.5± 0.4	−208.9	75.5	155.8	−0.3	59.5
		LS,s	780	2.90	30.2± 0.4	−184.6	76.3	133.7	−0.2	55.4
		LS,m	1095	3.24	25.0± 0.4	−168.2	76.7	120.0	−0.1	53.4
		LS,l	1788	3.81	18.0± 0.4	−146.1	77.2	102.5	−0.1	51.5
		BM,m	1095	3.24	55.4± 0.4	−212.9	68.4	143.2	−0.3	53.8
		BM,l	1788	3.81	53.3± 0.4	−210.1	67.7	143.2	−0.3	53.8
	Water	LS,ss	526	2.53	−300.8± 0.6	−76.0	77.6	0.0	−0.3	−299.5
		LS,s	800	2.90	−310.6± 0.7	−66.6	78.3	0.0	−0.2	−299.1
		LS,m	1132	3.25	−318.6± 0.7	−59.7	78.9	0.0	−0.1	−299.5
		LS,l	1826	3.81	−327.0± 0.7	−51.2	79.1	0.0	−0.1	−299.2
		BM,m	1132	3.25	−299.8± 0.6	−77.2	77.0	0.0	−0.3	−300.3
		BM,l	1826	3.81	−301.5± 0.6	−75.6	79.0	0.0	−0.3	−298.4

**Figure 3:**
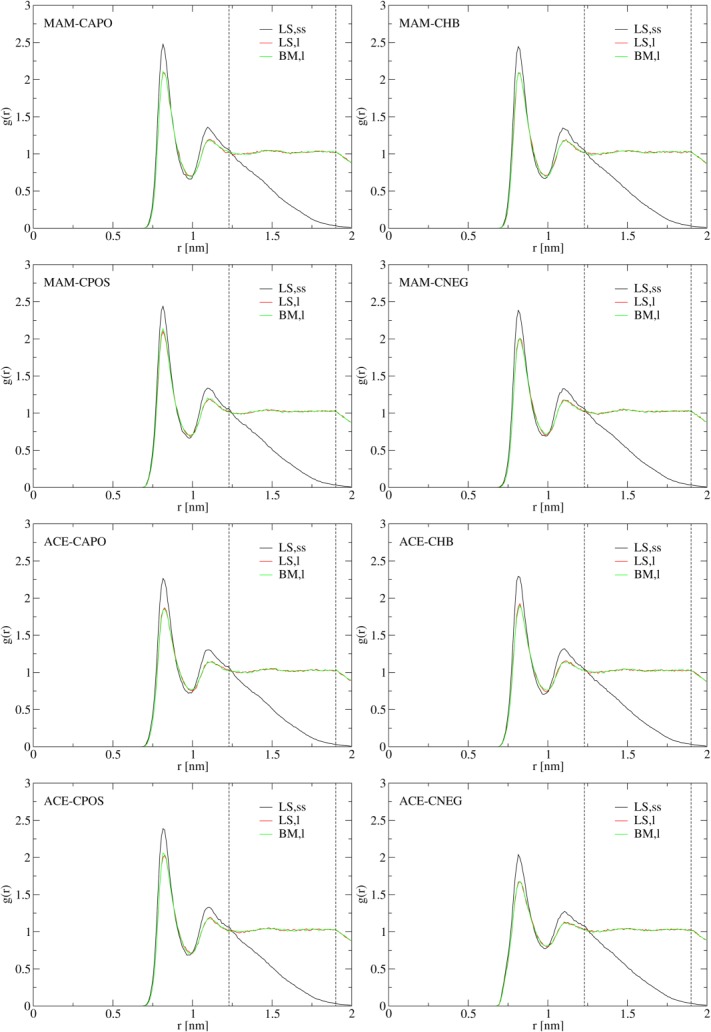
Radial distribution *g*(*r*) [eq. (2)] of water oxygen atoms around the center of mass of the 60 buckyball carbon atoms, evaluated from simulations LS,ss, LS,l, and BM,l for systems containing guest molecules MAM or ACE in hydrated host molecules CAPO, CHB, CPOS, or CNEG. The vertical dashed lines indicate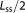 and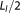, that is, the threshold beyond which *g*(*r*) decays due to box-corner artifacts.

**Figure 4:**
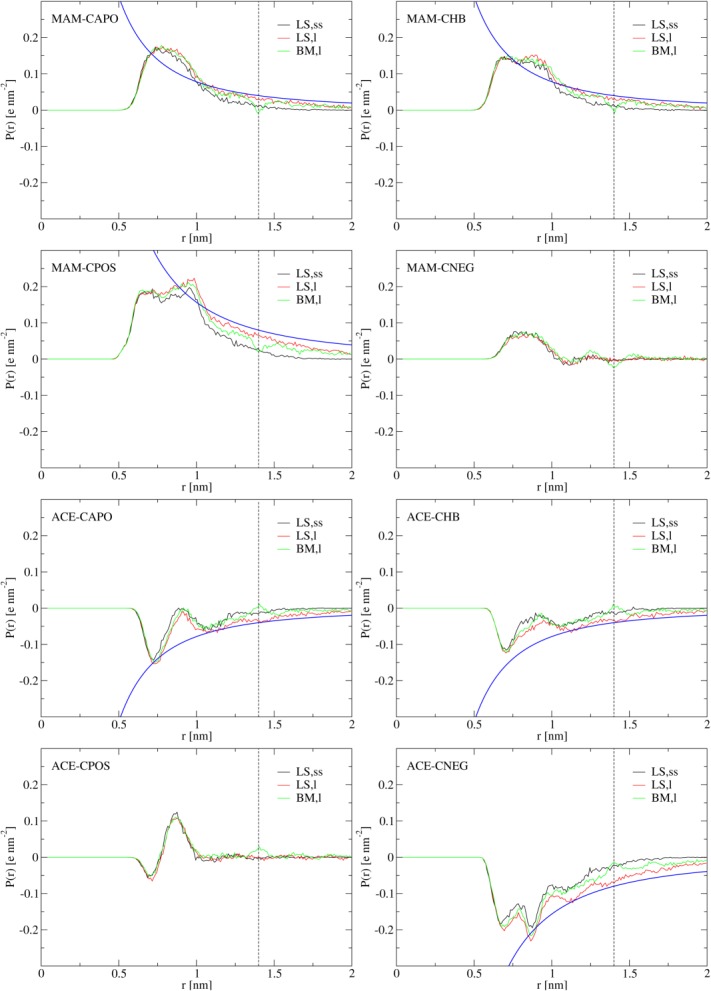
Radial polarization *P*(*r*) [eq.(3)] of water molecules around the MAM nitrogen atom or the ACE carboxylate carbon atom in hydrated host molecules CAPO, CHB, CPOS, or CNEG, evaluated from simulations LS,ss, LS,l, and BM,l. The blue line depicted for systems MAM-CAPO, MAM-CHB, MAM-CPOS, ACE-CAPO, ACE-CHB, and ACE-CNEG is the Born polarization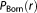 [eq. (6)] according to a system where the total solute (guest and host) charge is centered at the MAM nitrogen or the ACE carboxylate carbon atom. The vertical dashed line indicates the cutoff distance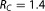 nm used in simulations BM,l.

For all guest-host complexes, in simulations LS,ss, *g*(*r*) shows spurious density fluctuations (overestimated height of the first and second peaks; Fig. 3) and *P*(*r*) shows marked underpolarization in comparison to the Born polarization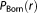 (due to the large influence of periodic solute copies, a consequence of the small edge length of the computational box; Fig. 4). As discussed in Appendix section “*Calculation of*
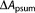
”, the former artifact might cause the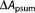 correction [eq. (14)] to be inadequate. The long-range regime of the latter artifact is corrected by the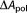 correction [eqs. (10) and (11)]. However, short-range artifacts in the solvent polarization, which affect solvation shell structure in the vicinity of the guest-host complex, cannot be represented by a continuum-electrostatics description of the solvent and are thus not captured by the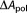 correction [eqs. (10) and (11)]. Such artifacts appear especially pronounced in simulation LS,ss of complexes MAM-CNEG and ACE-CNEG. This is evidenced by the relatively large deviation of the short-range *P*(*r*) in simulation LS,ss from the corresponding polarization in simulations LS,l and BM,l in the fully-charged complex ACE-CNEG (Fig. 4) and in complex MAM-CNEG containing the uncharged guest molecule (data not shown). Note furthermore that in simulations BM,m and BM,l, *P*(*r*) shows marked cutoff artifacts at the cutoff distance of 1.4 nm (a dip in the case of MAM-containing complexes and a crest in the case of ACE-containing complexes) and just before the cutoff distance (overpolarization in the case of MAM-containing complexes). These peculiarities, arising from molecule-based cutoff truncation in an explicit-solvent system, are also not captured by the continuum-electrostatics analog of *P*(*r*) for the BM scheme^94^,^97^ and can thus not be remedied by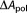.

For the systems considered in the present study, the magnitude of correction terms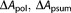, and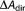 (CPOS and CNEG only) is very large (on the order of 50–200 kJ mol^−1^). For hydration in pure water,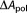 is always negative (independent of the sign of the guest charge) to account for the underhydration of the guest molecule caused by the presence of neighboring periodic copies (LS scheme), or the omission of guest-solvent interactions outside the cutoff sphere (BM scheme). In contrast,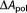 is positive for MAM-charging in CNEG and ACE-charging in CPOS because in these complexes the initial state of the TI procedure contains a charged guest-host complex, whereas the final state contains a neutral complex, that is, the electrostatic potential sampled at the guest atom sites is spurious in the initial rather than in the final state. As is the case for hydration in pure water,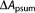 is negative for the charging of cations (MAM) and positive for the charging of anions (ACE). Because of the absence of electrostatic guest-host interactions,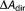 is zero for the charging in CAPO. As the LS and BM electrostatic interaction functions used in the present study are reduced in comparison to the Coulombic component (presence of self- and reaction-field terms^71^,^86^),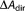 is negative for charging of MAM in CHB and the oppositely-charged CNEG and of ACE in CHB and the oppositely-charged CPOS. Likewise, it is positive for charging of the guests in the like-charged hosts, that is, MAM in CPOS and ACE in CNEG. In comparison to the other correction terms,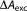 is of rather small magnitude (0.1–0.4 kJ mol^−1^), because it is a short-range electrostatic interaction reduced by the short-range singularity associated with the Coulombic component. For the LS scheme, the magnitude of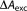 decreases with increasing box-edge length due to decreasing periodicity artifacts. For the BM scheme,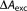 is independent of box-edge length.

Table3 reports raw free energies of charging the guest molecule due to the host and periodic host copies (LS scheme only),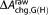 [eq. (22)], or due to the solvent and periodic solvent copies (LS scheme only),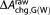 [eq. (23)], and corresponding corrected values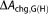 and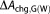 [eqs. (24) and (25)].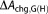 differs from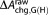 in that it is exempt of interaction of the guest with periodic host copies (LS scheme) or of reaction-field terms (BM scheme) and corrected to have Coulombic electrostatic interactions between the guest and the host within the central computational box (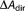; Table2).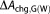 differs from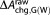 in that it is corrected for all solvent-associated artifacts, that is, spurious solvent polarization and wrong dielectric permittivity of the solvent model, improper electrostatic potential summation and the presence of electrostatic interactions between excluded atoms (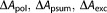; Table2). It can be seen that application of the correction terms may cause considerable shifts in the ratio of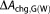 and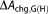. In particular, the relative dominance of the components reverses in the case of MAM-CHB and MAM-CPOS, that is, while interactions with the host dominate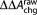, those with the solvent dominate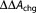 (Table3). Moreover, in the case of ACE-CPOS, the signs of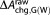 and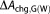 are different for simulations LS,ss, LS,s, LS,m, BM,m, and BM,l. Corresponding uncorrected values are slightly negative, that is, indicative of favorable ACE-solvent interactions, whereas the corrected values are positive, indicative of a solvent polarization unfavorable for interactions with an anion (solvent polarized by the positively-charged functional group in CPOS, which is located closer to the solvent than is the ACE ion). For complexes MAM-CNEG, ACE-CPOS, and ACE-CNEG, the raw and corrected free energies of charging due to the host molecule,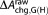 and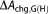, are the dominant components in the charging contributions to the respective binding free energies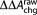 and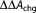, whereas for complex ACE-CHB the charging contributions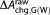 and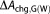 due to the solvent are dominant.

**Table 3:** Charging free-energy contributions due to the solvent, 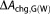 [eq. (25)], and due the host molecule,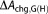 [eq. (24)], of the guest molecules MAM and ACE in hydrated host molecules CAPO, CHB, CPOS, and CNEG (section “MD simulations”). Values obtained with the LS scheme in boxes of edge lengths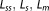, and *L*_*l*_ are labeled LS,ss, LS,s, LS,m, and LS,l, respectively, and values obtained with the BM scheme in boxes of edge lengths *L*_*m*_ and *L*_*l*_ are labeled BM,m and BM,l, respectively (section “Simulated guest-host systems”). The charging free energies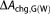 and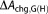 are calculated as the sum of the raw charging free energy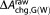 [eq. (23)] and the correction terms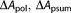, and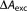 [eqs. (10), (11), (14), (15), and (18)] and as the sum of the raw charging free energy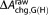 [eq. (22)] and the correction term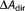 [eqs. (16) and (17)], respectively (section “Solute and solvent contributions to the free energy of charging”).

Guest	Host	Scheme	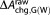 (kJ mol^−1^)	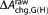 (kJ mol^−1^)	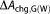 (kJ mol^−1^)	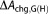 (kJ mol^−1^)
MAM	CAPO	LS,ss	−4.4	0.0	−151.2	0.0
		LS,s	−12.4	0.0	−151.2	0.0
		LS,m	−17.6	0.0	−151.4	0.0
		LS,l	−24.7	0.0	−151.1	0.0
		BM,m	−8.5	0.0	−150.0	0.0
		BM,l	−9.7	0.0	−149.1	0.0
	CHB	LS,ss	−1.8	−36.9	−147.9	−37.6
		LS,s	−9.9	−37.4	−148.1	−37.8
		LS,m	−15.0	−38.2	−148.5	−38.5
		LS,l	−21.9	−37.8	−148.2	−38.0
		BM,m	−6.4	−37.7	−146.9	−38.6
		BM,l	−7.4	−36.9	−145.9	−37.8
	CPOS	LS,ss	−48.5	164.2	−333.8	320.0
		LS,s	−75.1	186.3	−336.1	319.6
		LS,m	−91.6	199.2	−337.2	319.2
		LS,l	−114.5	217.3	−338.1	319.9
		BM,m	−54.7	176.9	−336.9	319.3
		BM,l	−57.0	176.7	−335.4	319.1
	CNEG	LS,ss	57.3	−291.5	51.6	−449.4
		LS,s	69.2	−314.6	54.3	−449.2
		LS,m	76.2	−328.8	55.6	−449.3
		LS,l	85.2	−346.4	56.5	−449.3
		BM,m	55.4	−304.3	57.6	−449.4
		BM,l	55.7	−304.4	57.5	−449.5
ACE	CAPO	LS,ss	−74.5	0.0	−70.2	0.0
		LS,s	−84.5	0.0	−70.5	0.0
		LS,m	−90.8	0.0	−70.6	0.0
		LS,l	−99.1	0.0	−71.1	0.0
		BM,m	−65.9	0.0	−69.6	0.0
		BM,l	−66.2	0.0	−69.9	0.0
	CHB	LS,ss	−71.1	−46.7	−66.2	−46.9
		LS,s	−81.1	−46.9	−66.7	−47.0
		LS,m	−87.2	−46.9	−66.7	−47.0
		LS,l	−95.4	−47.3	−67.1	−47.4
		BM,m	−62.8	−46.7	−65.6	−47.0
		BM,l	−63.1	−46.9	−65.8	−47.2
	CPOS	LS,ss	−16.1	−272.6	129.1	−430.2
		LS,s	−6.4	−295.5	131.3	−430.0
		LS,m	−0.4	−309.7	132.1	−430.2
		LS,l	7.2	−327.0	133.2	−430.0
		BM,m	−5.2	−285.3	133.6	−430.1
		BM,l	−4.0	−285.3	133.4	−430.2
	CNEG	LS,ss	−134.5	172.0	−268.2	327.8
		LS,s	−163.8	194.0	−272.3	327.7
		LS,m	−182.5	207.5	−274.1	327.5
		LS,l	−207.1	225.1	−276.1	327.6
		BM,m	−129.0	184.4	−273.8	327.6
		BM,l	−131.1	184.4	−273.8	327.6

The most drastic change in contributions to the binding free energy occurs with MAM-CPOS and is effected by the large value of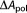 (−146.4 to −212.8 kJ mol^−1^; Table2) in combination with the fact that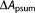 also has a negative sign (cation charging) [eqs. (14) and (15)]. The least change in contributions to the binding free energy occurs with ACE-CHB and is effected by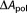 and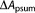 approximately canceling each other (−48.8 to −71.0 kJ mol^−1^ for the former versus 67.6 to 77.2 kJ mol^−1^ for the latter; Table2) by virtue of the positive sign of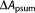 in the case of anion charging [eqs. (14) and (15)]. Note, in this context, that in system MAM-CPOS,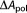 corrects for spurious polarization around charges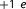 and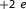 in the initial and final states of the TI, respectively. Thus, considering, for example, the LS scheme,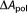 approximately evaluates to three times the correction for artificial periodicity in the case of charging a single monovalent ion in a box of edge length *L*,^23^,^54^,^92^ namely to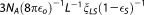, where the factor three arises from the proportionality of this correction to the square of the ionic charge (Appendix section “*Calculation of*
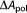
”) [eq. (28)].

The raw charging free energies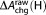 and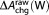, as well as the corrected data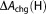 and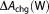 may be used to calculate raw and corrected charging contributions to the binding free energies,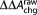 and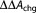, respectively [eqs. (1) and (21)]. For the corrected, that is, methodology-independent data, this can be done for all possible combinations of system sizes or/and electrostatics schemes used in the simulations of charging in water and in the host molecule. In practice, binding free energies are often calculated using computational boxes that are smaller for the in-water than for the in-host simulations. Table4 reports the uncorrected data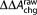 for such a situation (in-water charging in small box size, here *L*_*ss*_ for the LS scheme and *L*_*m*_ for the BM scheme; in-host charging in large box size, here *L*_*l*_ for the LS and BM scheme) and for those situations where approximate cancelation of periodicity-induced artifacts is expected to occur (in-water and in-host charging in boxes of equal size). Note, however, that the latter cancelation is of greater relevance for the LS scheme, because raw charging free energies obtained from simulations with the BM scheme are less sensitive to system size.^89^ The averages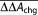 of corrected values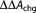 [eq. (21)] over all combinations of box sizes used for in-water and in-host charging, along with associated root-mean-square deviations are also provided. Values obtained for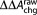 based on *L*_*ss*_ for in-water charging and *L*_*l*_ for in-host charging differ by −28.1, −28.1, −35.2, and −21.0 kJ mol^−1^ for MAM binding to CAPO, CHB, CPOS, and CNEG, respectively, and by −27.0, −27.5, −19.7, and −35.5 kJ mol^−1^ for ACE binding to CAPO, CHB, CPOS, and CNEG, respectively, from corresponding data for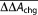 (LS scheme), and values obtained for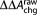 based on *L*_*m*_ for in-water charging and *L*_*l*_ for in-host charging differ by −15.8, −15.4, −19.0, and −12.4 kJ mol^−1^ for MAM binding to CAPO, CHB, CPOS, and CNEG, respectively, and by 4.0, 3.2, 7.8, and −0.1 kJ mol^−1^ for ACE binding to CAPO, CHB, CPOS, and CNEG, respectively, from corresponding data for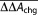 (BM scheme). The majority of these deviations are non-negligible, and it is thus essential to correct raw charging contributions to binding free energies. Note that box-edge length dependence is more pronounced for simulations with the LS scheme, because here the system-size parameter crucially determines the magnitude of artificial periodicity artifacts.

**Table 4:** Raw charging contributions 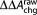 [eq. (1)] to binding free energies of guest molecules MAM and ACE to hydrated host molecules CAPO, CHB, CPOS, and CNEG based on values for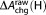 and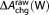 calculated in four different system sizes (boxes of edge lengths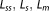, and *L*_*l*_; section “Simulated guest-host systems”) using LS or BM electrostatic interactions (Table2). Only a subset of the 16 (LS scheme-based) or four (BM scheme-based) possible combinations is reported.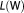 and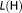 denote the box-edge lengths used for simulations of in-water and in-host charging, respectively. For comparison, averages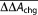 of corrected values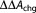 [eq. (21)] over all 16 combinations of box sizes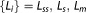 and *L*_*l*_ in the case of the LS scheme, over all four combinations of box sizes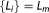 and *L*_*l*_ in the case of the BM scheme, or over the union of the two latter sets (denoted LS+BM) used for in-water and in-host charging, along with associated root-mean-square deviations (rmsd) are also provided.

Guest			MAM				ACE			
Host			CAPO	CHB	CPOS	CNEG	CAPO	CHB	CPOS	CNEG
Scheme	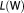	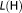	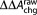 (kJ mol^−1^)							
LS	*L*_*ss*_	*L*_*l*_	147.9	112.9	275.4	−88.6	201.7	158.1	−19.0	318.8
LS	*L*_*ss*_	*L*_*ss*_	168.2	133.9	288.3	−61.6	226.3	183.0	12.1	338.3
LS	*L*_*s*_	*L*_*s*_	169.4	134.5	293.0	−63.6	226.1	182.6	8.7	340.8
LS	*L*_*m*_	*L*_*m*_	170.5	134.9	295.7	−64.5	227.8	184.5	8.5	343.6
LS	*L*_*l*_	*L*_*l*_	173.1	138.1	300.6	−63.4	227.9	184.3	7.2	345.0
BM	*L*_*m*_	*L*_*l*_	164.0	129.4	293.4	−75.0	233.6	189.8	10.5	353.1
BM	*L*_*m*_	*L*_*m*_	165.2	129.6	295.9	−75.2	233.9	190.3	9.3	355.2
BM	*L*_*l*_	*L*_*l*_	165.8	131.2	295.2	−73.2	235.3	191.5	12.2	354.8
			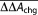 (kJ mol^−1^)							
LS	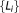	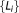	176.0	141.0	310.6	−67.6	228.7	185.6	0.7	354.3
BM	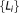	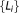	179.8	144.8	312.4	−62.6	229.6	186.6	2.7	353.2
LS+BM	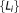	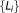	176.7	141.8	310.9	−66.6	228.9	185.8	1.1	354.1
			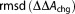 (kJ mol^−1^)							
LS	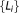	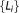	0.8	0.9	1.9	2.0	0.4	0.5	1.6	3.0
BM	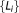	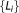	1.4	1.6	1.5	1.4	1.0	1.0	1.0	1.0
LS+BM	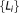	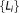	1.8	1.9	2.0	2.8	0.6	0.8	1.7	2.7

If both the in-water and in-host charging simulations are conducted in boxes of identical edge length, the deviations are significantly reduced for the LS scheme, that is, they evaluate to −2.9, −2.9, −10.0, and 4.2 kJ mol^−1^ for MAM binding to CAPO, CHB, CPOS, and CNEG, respectively, and to −0.8, −1.3, 6.5, and −9.3 kJ mol^−1^ for ACE binding to CAPO, CHB, CPOS, and CNEG, respectively, based on simulations in boxes of edge length *L*_*l*_, the best agreement with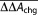 thus being achieved for complexes containing the apolar CAPO and CHB host molecules. Note that simulations in equisized boxes do not lead to an improvement for the BM scheme, where, using data pertaining to edge length *L*_*l*_, the deviations of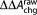 from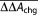 are −14.0, −13.6, −17.2, and −10.6 kJ mol^−1^ for MAM binding to CAPO, CHB, CPOS, and CNEG, respectively, and 5.7, 4.9, 9.5, and 1.6 kJ mol^−1^ for ACE binding to CAPO, CHB, CPOS, and CNEG, respectively.

The averages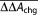 differ for simulation data pertaining to solely the LS or BM scheme by 0.9–5.0 kJ mol^−1^. Overall, the averages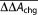 based on the BM scheme data differ on average by 2.4 kJ mol^−1^ from the LS scheme data, the agreement between the two different electrostatics schemes being better for ACE-containing complexes (average absolute difference 1.3 kJ mol^−1^) than for MAM-containing complexes (average absolute difference 3.6 kJ mol^−1^). This might be due to favorable cancelation of artifacts in the ACE-containing complexes, as well as the more pronounced cutoff artifacts in *P*(*r*) and the continuum-electrostatics-based correction scheme insufficiently capturing the pronounced overpolarization within the cutoff sphere for the MAM-containing complexes (Fig. 4). In comparison to the polarization in a homogeneous dielectric medium, approximated here by the Born polarization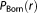 [eq. (6)] around a charge of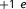 (MAM-CAPO, MAM-CHB),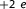 (MAM-CPOS),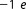 (ACE-CAPO, ACE-CHB), or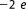 (ACE-CNEG) centered at the MAM nitrogen or the ACE carboxylate carbon atom, hydration shell peaks in *P*(*r*) appear more pronounced for MAM in comparison to ACE in neutral host cavities CAPO and CHB and significantly broader for MAM in comparison to ACE in host cavities CAPO, CHB and the like-charged functionalized one (CPOS in the case of MAM, CNEG in the case of ACE). A less pronounced water radial polarization around anionic in comparison to cationic solutes was also observed before in the context of the hydration of monoatomic ions and can be drawn back to a decreased orientational freedom of water molecules around cations.^58^

The corrected charging contributions (entailing all possible combinations of box sizes for in-water and in-host charging) show rmsd values within 2.5 kJ mol^−1^ for all complexes except MAM-CNEG (2.8 kJ mol^−1^) and ACE-CNEG (2.7 kJ mol^−1^). As discussed above, the spread in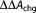 for these systems may be drawn back to the inability of the continuum-electrostatics approximation to capture short-range artifacts in solvent structure which appear to be very strong for the LS,ss simulations of these complexes.

Both raw and corrected charging contributions to the binding free energy,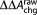 and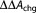 (Table4), obey intuitive reasoning in that they are least favorable for the like-charged guest-host complexes (MAM-CPOS, ACE-CNEG), considerably less unfavorable for the apolar host cavity (CAPO) and the host cavity allowing hydrogen bonding (CHB) and least unfavorable for the oppositely-charged guest-host complexes (MAM-CNEG, ACE-CPOS). In comparison to charging the guest in water, binding to the host is, however, only favorable in the case of MAM-CNEG (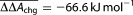; Table4). The charging of guest molecule ACE is basically indifferent toward pure water or host CPOS environments (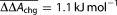; Table4), which can probably be explained in terms of water being an extremely good solvent for anion solvation because the hydrogen atoms of the water molecule can approach anions very closely.^98^–^101^

Altogether, as it can significantly alter the charging contribution to binding free energies and thus crucially change the interpretation or prediction of experimental data, analysis of possible electrostatic artifacts and application of required correction terms appears very important and should be considered an integral part of careful free-energy calculation studies if changes in the net charge are involved. Note that for more complex guest-host systems (e.g., a drug-receptor complex) it might be necessary to take into account the possible flexibility of the molecules, giving rise to time-dependent correction terms.

## Conclusion

The calculation of binding free energies of charged species to a target molecule is a frequently encountered problem in MD studies of (bio-)chemical thermodynamics. A number of important endogenous receptor-binding molecules (e.g., glutamate, acetylcholine), enzyme substrates (e.g., superoxide anion, lysine) or drug molecules (e.g., aspirin, proguanil) have a nonzero net charge. Absolute binding free energies, as well as binding free energies relative to another molecule with a different net charge will be affected by artifacts due to the used effective electrostatic interaction function and associated parameters (e.g., size of the computational box). This is increasingly being recognized in the field of free-energy simulations. Independently from the authors' work, Rocklin et al. proposed a very similar correction scheme.^102^ In the present study, charging contributions to binding free energies of either of two ionic guest molecules, MAM and ACE, to functionalized buckyball-like host cavities were calculated with classical atomistic MD simulation. Electrostatic interactions were treated using a LS scheme or a BM scheme, and the simulations were conducted in boxes of four different edge lengths. It was illustrated that: (i) the charging free energies of the guest molecules in water and in the host molecule strongly depend on the applied methodology; (ii) the charging free energies of the guest molecules in water and in the host molecule obtained from the LS scheme present a non-negligible dependence on the edge length of the simulation box; (iii) considering the investigated systems, error cancellation in computed charging contributions to binding free energies is only approximately guaranteed for systems with an apolar cavity (zero host charges) if corresponding in-water and in-host charging simulations are performed with the LS scheme in equisized boxes; (iv) neglect of correction terms for the artifacts introduced by the finite size of the simulated system and the use of an effective electrostatic interaction function considerably impairs the thermodynamic interpretation of guest-host interactions, and in particular the relative contributions of the solvent and the host compound; (v) application of correction terms for spurious solvent polarization and wrong dielectric permittivity of the solvent model, improper electrostatic potential summation, effective guest-host direct electrostatic interactions, and the presence of electrostatic interactions between excluded solute atoms in the Hamiltonian yields consistent results for the charging contribution to binding free energies. In particular, rmsd values over 20 results lie within 2.5 kJ mol^−1^ for all systems except MAM-CNEG and ACE-CNEG. For these systems, the spread might be drawn back to strong artifacts in solvent configurational sampling in a very small computational box using the LS scheme for the treatment of electrostatic interactions that are not captured by the continuum-electrostatics-based correction procedure.

As long as simulations of macroscopic nonperiodic systems with Coulombic electrostatic interactions, or electrostatic interactions truncated at sufficiently large distances, such that an adequate representation of experimental bulk systems is achieved, are out of reach, the proposed correction scheme for the charging contribution to binding free energies is a crucial step in obtaining thermodynamically sensible results for the free energy of binding of charged ligands.

## APPENDIX

### Calculation of

Equation (12) describes calculation of a charging free energy via TI of the environment-generated electrostatic potential monitored at the guest atoms along a scaling parameter λ which grows the guest atomic partial charges from zero to the full charge state. This integration is commonly performed numerically using a finite number of intermediate guest charge states. In the present work, six charge states were used (section “*Free-energy correction terms*”). Evaluation of requires conduction of two [eq. (10)] or four [eq. (11)] such TIs. However, three alternative computationally less intensive approaches may be conceived of:

1. As the electrostatic potential at the guest atoms varies linearly with the guest charge state, the TI can be cut down to involve only the initial and final state, that is, eq. (12) may be simplified to
  A1
  where the factor (1/2) arises from numerical integration using the trapezoidal rule and the electrostatic potentials are evaluated at (uncharged guest) and (fully charged guest). then follows from eqs. (10) and (11).
2. A linear variation of the electrostatic potential at the guest atoms with the guest charge state allows in principle a linear-response approximation according to
  A2
  where the factor (1/2) arises from the assumption of linear response, and the second term in the curly brackets accounts for a possibly nonzero host charge *Q*_*H*_. In the latter case, eq. (27) is very approximate because it assumes that the host charge is concentrated at one point and experiences the average of electrostatic potentials monitored at the guest atom sites. then follows from eqs. (10) and (11).
3. A crude approximation to for the LS scheme may be obtained through usage of the analytical formula pertinent to the case of solvation of a single nonpolarizable monoatomic ion of radius *R*_*I*_ in a box of edge length *L* filled with a homogeneous dielectric medium of permittivity.^54^,^92^ In the present case of a solvated guest-host system, this formula may be rewritten
  A3
  where.^82^,^83^,^103^ Equation (28) approximates the guest-host complex by a cavity of radius *R*_*vdW*_ containing a point charge of magnitudes *Q*_*H*_ and in the initial and final states, respectively.

As an example, the case of MAM charging in CPOS in system LS,l is considered. Equation (12), using six charge states as described in section “*Free-energy correction terms*” yields (Table2). Equation (26) yields the same result,. Equation (27) yields, with contributions of −97.9 and −49.4 kJ mol^−1^ from the first and second term in curly brackets, respectively. Using and, eq. (28) yields −149.4 kJ mol^−1^. Whereas eq. (26) is numerically accurate (due to a linear charging profile the initial and final points of the charging curve are sufficient to calculate the charging free energy), eqs. (27) and (28) rely on the afore mentioned approximations, which causes the resulting value to be less accurate. However, both of the two latter estimates deviate less than from the numerically accurate result.

### Calculation of

In Ref.^61^, was calculated as eq. (14) for the LS scheme and using

A4

for the BM scheme. is an estimate for the volume of the solute cavity (guest molecule in the case of in-water charging; host molecule in the case of in-host charging). Here, the volume of the host cavity was approximated by a sphere of radius *R*_*vdW*_ (Table5).

**A1:** Averages  and root-mean-square deviations (rmsd) of corrected values [eq. (20) and Table2], for charging of the guest molecules MAM and ACE in hydrated host molecules CAPO, CHB, CPOS, and CNEG (section “MD simulations”). The correction term was evaluated using eq. (14) for the LS scheme and either eq. (29) or (15) for the BM scheme. The volume *V*_*vdW*_ of the host cavity was approximated by a sphere of radius *R*_*vdW*_ [eq. (29)]. For a given approach and choice of *R*_*vdW*_, averages  over the individual rmsd values are provided.

Guest	Host	Equations (14) and (29), *R*_vdW_ = 0.5 nm		Equations (14) and (29), *R*_vdW_ = 0.6 nm		Equations (14) and (29), *R*_vdW_ = 0.7 nm		Equations (14) and (15)	
		(kJ mol^−1^)	rmsd (kJ mol^−1^)	(kJ mol^−1^)	rmsd (kJ mol^−1^)	(kJ mol^−1^)	rmsd (kJ mol^−1^)	(kJ mol^−1^)	rmsd (kJ mol^−1^)
MAM	CAPO	−152.1	1.3	−151.5	0.4	−150.6	0.9	−150.6	0.8
	CHB	−187.0	1.4	−186.4	0.7	−185.5	1.1	−185.6	1.0
	CPOS	−18.1	2.5	−17.5	1.9	−16.6	1.5	−16.7	1.5
	CNEG	−395.3	1.8	−394.7	1.6	−393.8	2.1	−393.8	2.1
ACE	CAPO	−68.7	2.7	−69.3	1.8	−70.2	0.6	−70.3	0.5
	CHB	−111.9	2.7	−112.5	1.8	−113.4	0.6	−113.4	0.6
	CPOS	−296.5	3.3	−297.1	2.5	−298.0	1.6	−298.0	1.6
	CNEG	56.1	2.9	55.5	2.5	54.6	2.5	54.6	2.5
(kJ mol^−1^)			2.3		1.7		1.4		1.3

In the present study, a slightly different equation was used for [eq. (15)] (section “*Free-energy correction terms*”). Equation (15) derives from expressing the product of the fraction *f*_*c*_ of the cutoff sphere occupied by water with the water number density in the original expression^54^,^89^ for in the case of hydration of a single monoatomic ion,

A5

as

A6

Similarly, eq. (14) derives from expressing the product of the fraction *f*_*b*_ of the computational box occupied by water with the water number density in the original expression^54^,^89^ for in the case of hydration of a single monoatomic ion,

A7

as

A8

Note that the expressions for the water number density used in eqs. (14) and (15), that is, and, respectively, might not be adequate for simulations LS,ss, because of spurious water density fluctuations in this very small system that are not captured by a global (box-volume associated) density measure. In particular, for simulations LS,ss, the height of the first and second hydration shell peaks around the buckyball cavity is overestimated by up to 18 and 15% for MAM and ACE, respectively, in hydrated host molecules CAPO, CHB, CPOS, and CNEG in comparison to simulations LS,l and BM,l (Fig. 3). Equation (15) seems advantageous in comparison to eq. (29) because it yields greater methodological independence, as evidenced by the root-mean-square deviations (rmsd) of corrected charging free energies [eq. (20)] for charging the guest molecules in the host, reported in Table5, and is independent of the volume of the solute. Three different choices for *R*_*vdW*_ were investigated, namely, 0.6, or 0.7 nm. Distances of 0.5 and 0.7 nm from the center of mass of the 60 buckyball carbon atoms correspond to the average location of the 60 buckyball carbons atoms and the value where the water oxygen radial distribution function starts to deviate from zero, respectively. Overall, the cavity volume-independent eqs. (14) and (15) achieve lower rmsd values, the average rmsd over all systems being 1.3 kJ mol^−1^, as compared to 2.3, 1.7, and 1.4 kJ mol^−1^ for the three above choices of radii in eq. (29).
